# Prof. E. S. Carr

**Published:** 1868-11-15

**Authors:** 


### *EDITORIAL.*

***Prof. E. S. Carr,***

Of Madison, Wisconsin, whilom our preceptor in the beautiful science of Chemistry, and known in honor and high esteem by old graduates and students of Castleton, Albany, Philadelphia, and Rush Medical College, we understand is about to visit and perhaps remove to San Francisco. We take the highest pleasure in commending him to the courtesies and friendship of all "to whom these letters may come" in the occidental and earthquake regions. Unsurpassed as a private instructor or public lecturer on Chemistry, Geology, and other Natural Sciences, he combines with this those genial qualities of heart which cement friendships never to be sundered.

